# Supplementary material for: Phenotypic Plasticity and Local Adaptation of Leaf Cuticular Waxes Favor Perennial Alpine Herbs under Climate Change
Source: Plants (Basel). 2021 Dec 31;11(1):120. doi: 10.3390/plants11010120 (PMC8747477; doi:10.3390/plants11010120)
Supplement: Supplementary file 1 [file plants-11-00120-s001.zip › plants-1433396-supplementary.pdf]

Supplementary Material

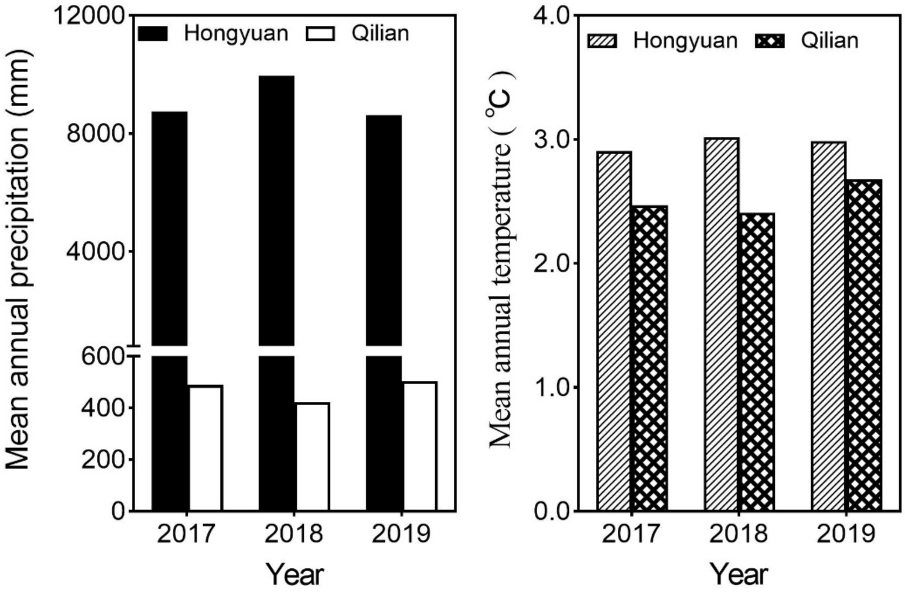

Figure S1. Histogram showing the mean annual precipitation and temperature of 2017, 2018 and 2019.

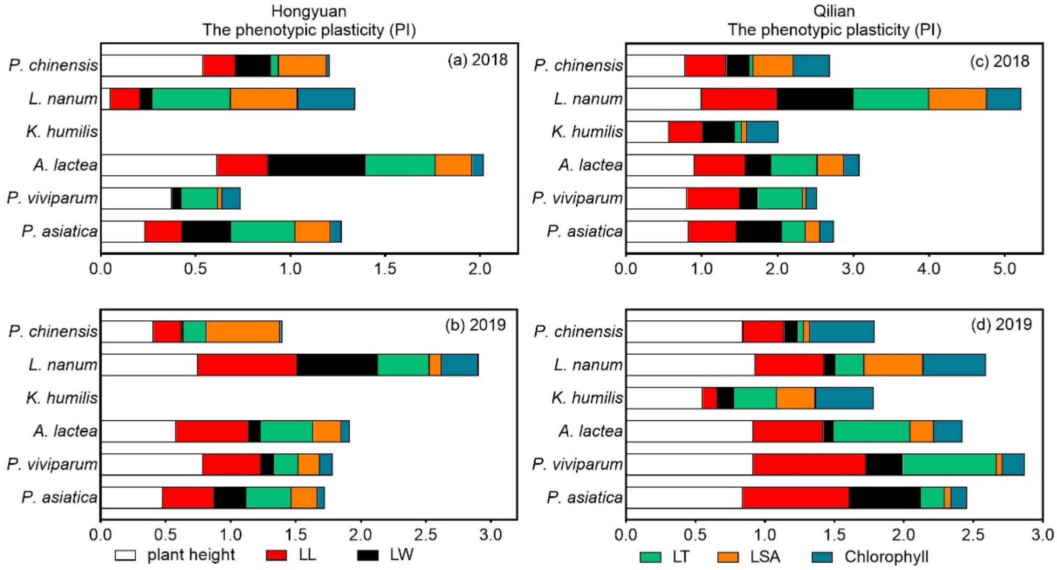

Figure S2. (a-d). Histogram showing the extent of phenotypic plasticity indices (PI) of phenotypic and chlorophyll traits

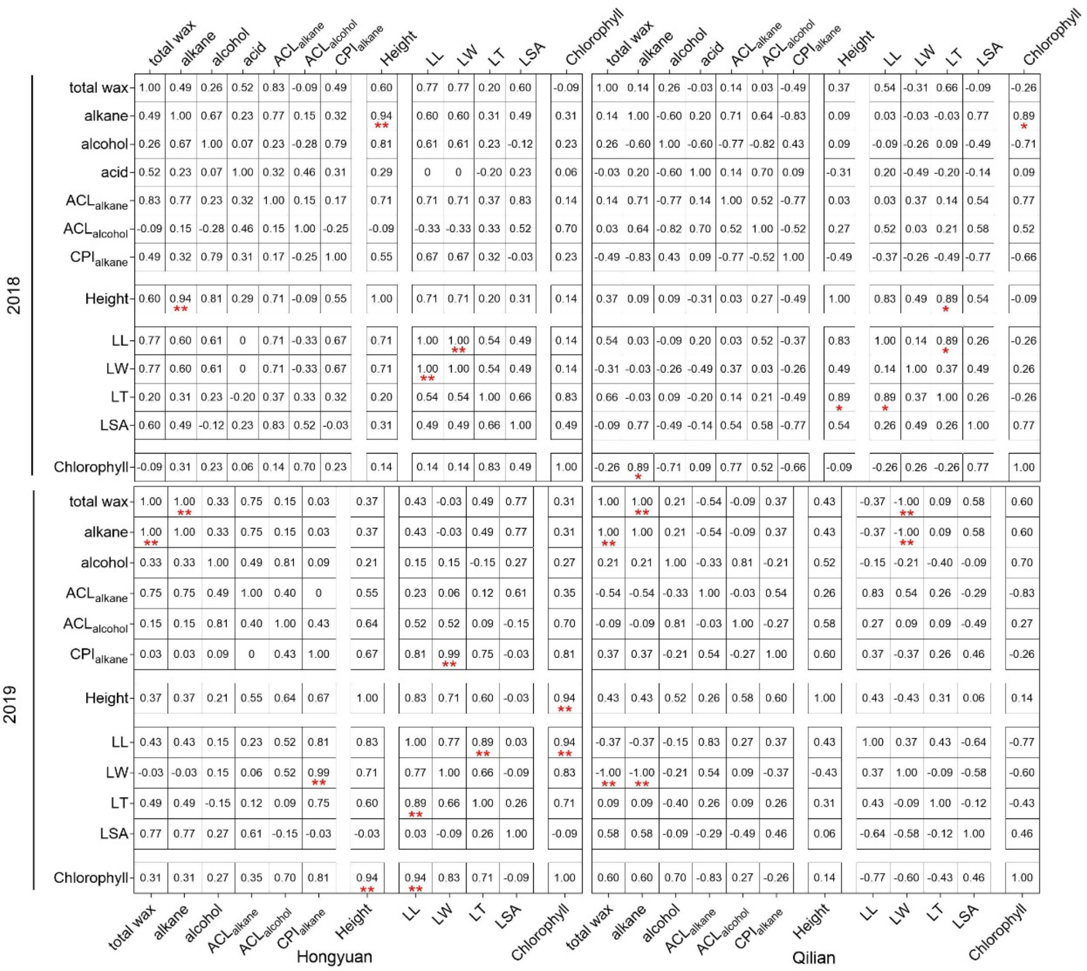

Figure S3. Spearman correlation analysis for leaf morphology and wax traits measured of different species transplant to Hongyuan and Qilian

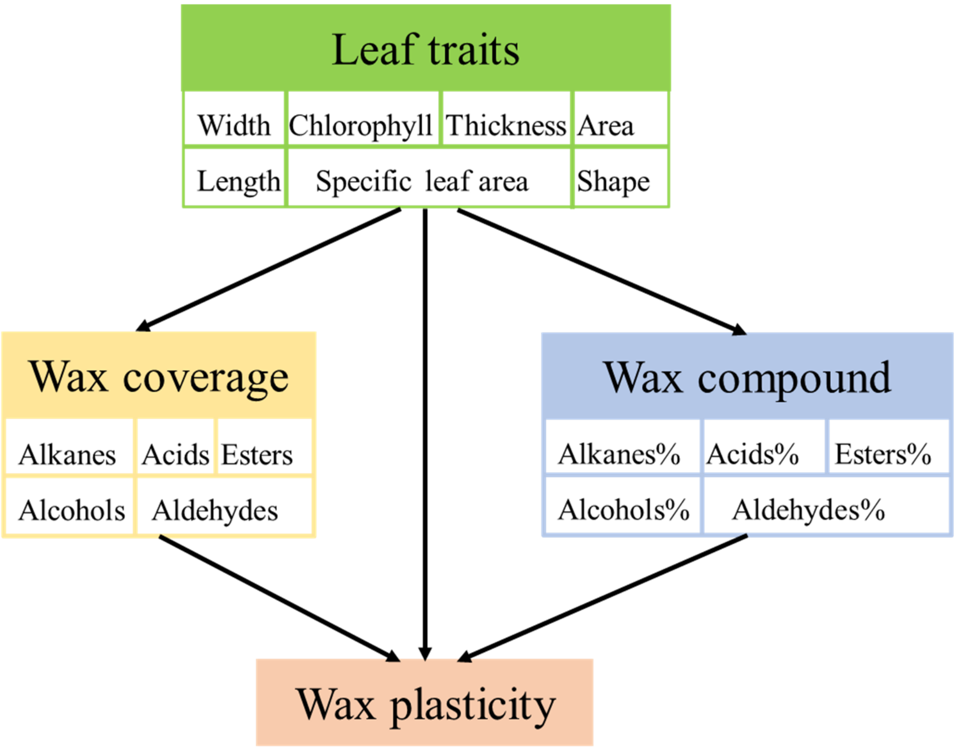

Figure S4. The a priori conceptual structural equation model (SEM) of the causal relationships among the leaf traits (i.e., length, width, thickness, area, shape, chlorophyll, specific leaf area), leaf epidermis wax (i.e., alkanes, acids, alcohols, esters, aldehydes), and wax phenotypic plasticity for each of the transplanting types (i.e., QL to HY, and HY to QL). All variables represent the alpine plant leaf temporal measurements of environmental changes. This a priori model was used for the multigroup comparisons among different transplanting types. The single-headed arrows indicate a hypothesized causal direct effect of one variable on another, and an indirect effect of one variable on another was exerted through other one or more variables.

Table S1. Soil properties and climate factors of Hongyuan and Qilian.

Parameters		Hongyuan	Qilian
Soil properties	Total nitrogen ($g·kg^{-1}$)	0.409	0.251
	Available nitrogen ($mg·kg^{-1}$)	0.035	0.029
	Total potassium ($g·kg^{-1}$)	1.665	0.731
	Available potassium ($mg·kg^{-1}$)	188.91	42.98
	Available phosphorus ($mg·kg^{-1}$)	131.34	46.14
	Organic matter ($g·kg^{-1}$)	374.02	236.12
	pH	6.21	8.48
Altitude (m)		3434	3701

Table S2. Summary of principal components analysis (PCA) of finall traits measured in 2019. Three principal components were kept according to the result of Horn's parallel analysis. Abbreviations: LL, leaf length; LW, leaf width; LT, leaf thickness; LSA, leaf specific area.

	Hongyuan		Qilian		
	PC1	PC2	PC1	PC2	PC3
Eigenvalues	4.152	3.464	4.322	2.205	1.485
% of Variance (var.)	46.13%	38.48%	48.02%	24.50%	16.50%
Cumulative % of var.	46.13%	84.61%	48.02%	72.52%	89.02%
Var.	PC1	PC2	PC1	PC2	PC3
Height	0.81	-0.36	-0.12	0.78	-0.61
LL	0.97	-0.21	-0.76	0.50	-0.34
LW	0.89	0.20	-0.91	-0.24	-0.22
LT	0.83	-0.34	-0.33	0.65	0.58
LSA	-0.26	-0.89	0.66	-0.02	0.18
Chlorophyll	0.92	0.10	0.89	-0.39	-0.14
total wax	0.01	-0.95	0.74	0.64	0.09
alkane	0.00	-0.95	0.77	0.54	0.08
alcohol	-0.39	-0.72	0.64	-0.08	-0.74
